# Biogenesis of Influenza A Virus Hemagglutinin Cross-Protective Stem Epitopes

**DOI:** 10.1371/journal.ppat.1004204

**Published:** 2014-06-12

**Authors:** Javier G. Magadán, Meghan O. Altman, William L. Ince, Heather D. Hickman, James Stevens, Aaron Chevalier, David Baker, Patrick C. Wilson, Rafi Ahmed, Jack R. Bennink, Jonathan W. Yewdell

**Affiliations:** 1 Laboratory of Viral Diseases, National Institute of Allergy and Infectious Diseases, National Institutes of Health, Bethesda, Maryland, United States of America; 2 Influenza Division, National Center for Immunization and Respiratory Diseases, Centers for Disease Control and Prevention, Atlanta, Georgia, United States of America; 3 Department of Biochemistry, University of Washington, Seattle, Washington, United States of America; 4 Department of Medicine, Section of Rheumatology, Committee on Immunology, Knapp Center for Lupus and Immunology Research, University of Chicago, Chicago, Illinois, United States of America; 5 Emory Vaccine Center, Department of Microbiology and Immunology, Emory University School of Medicine, Atlanta, Georgia, United States of America; Vanderbilt University Medical Center, United States of America

## Abstract

Antigenic variation in the globular domain of influenza A virus (IAV) hemagglutinin (HA) precludes effective immunity to this major human pathogen. Although the HA stem is highly conserved between influenza virus strains, HA stem-reactive antibodies (StRAbs) were long considered biologically inert. It is now clear, however, that StRAbs reduce viral replication in animal models and protect against pathogenicity and death, supporting the potential of HA stem-based immunogens as drift-resistant vaccines. Optimally designing StRAb-inducing immunogens and understanding StRAb effector functions require thorough comprehension of HA stem structure and antigenicity. Here, we study the biogenesis of HA stem epitopes recognized in cells infected with various drifted IAV H1N1 strains using mouse and human StRAbs. Using a novel immunofluorescence (IF)-based assay, we find that human StRAbs bind monomeric HA in the endoplasmic reticulum (ER) and trimerized HA in the Golgi complex (GC) with similar high avidity, potentially good news for producing effective monomeric HA stem immunogens. Though HA stem epitopes are nestled among several *N*-linked oligosaccharides, glycosylation is not required for full antigenicity. Rather, as *N*-linked glycans increase in size during intracellular transport of HA through the GC, StRAb binding becomes temperature-sensitive, binding poorly to HA at 4°C and well at 37°C. A *de novo* designed, 65-residue protein binds the mature HA stem independently of temperature, consistent with a lack of *N*-linked oligosaccharide steric hindrance due to its small size. Likewise, StRAbs bind recombinant HA carrying simple *N*-linked glycans in a temperature-independent manner. Chemical cross-linking experiments show that *N*-linked oligosaccharides likely influence StRAb binding by direct local effects rather than by globally modifying the conformational flexibility of HA. Our findings indicate that StRAb binding to HA is precarious, raising the possibility that sufficient immune pressure on the HA stem region could select for viral escape mutants with increased steric hindrance from *N*-linked glycans.

## Author Summary

Extensive variation in the IAV HA globular domain severely impedes influenza vaccination. Recent findings demonstrate that StRAbs, specific Abs to the highly conserved stem region of HA, can protect hosts against a broad variety of influenza virus strains. In investigating the binding of StRAbs to HA during its biogenesis in IAV-infected cells, we find that these Abs can bind HA monomers prior to their trimerization in the GC. Binding to HA becomes temperature-dependent, however, as *N*-linked oligosaccharides mature during transport of trimerized HA through the GC to the cell surface. Our findings support the potential use of monomeric HA stem immunogens to induce broadly neutralizing Abs, but raise the possibility of eventual viral escape from StRAbs, based on structural alterations in the HA that increase steric hindrance of HA stem *N*-linked glycans on StRAb binding.

## Introduction

IAV is responsible for considerable human morbidity and mortality, with enormous attendant economic costs. Current vaccines are at best effective only for a few years due to the evolution of human IAV strains that escape vaccine- or infection-induced immunity. The most relevant immune escape occurs in the HA, a homotrimeric viral surface type I integral membrane glycoprotein. HA initiates IAV infection by attaching virus to host cell sialic acid receptors and fusing viral and host membranes in acidifying endosomes. Abs to HA neutralize infectivity by blocking virus attachment or acid-triggered conformational changes on HA that mediate host-viral membranes fusion [1].

HA consists of a globular “head” domain containing the sialic binding site topping a stem containing the fusion domain [2]. Most neutralizing anti-HA Abs induced in experimental animals and humans are specific for the HA globular domain, which correlates well with the focus of the immune driven evolution on residues within this domain, particularly in the major antigenic regions (termed Sa, Sb, Ca1, Ca2, and Cb in H1 subtype HAs). Monoclonal Abs (mAbs) of defined fine-specificity have proven to be invaluable reagents for studying not only HA antigenicity [3]–[6] but also HA structure [7], function, and biogenesis in biochemical and immunohistochemical studies [8]–[10].

HA folding begins co-translationally [11], [12], and HA monomers are substantially folded in the ER [10]. HA trimerization occurs either in the ER [10], [13] or in a post-ER compartment [9] depending on the IAV strain studied. H1 HA oligomerization generates epitopes present in both the antigenic sites that bridge adjacent HA monomers (Ca1 and Ca2) as well as epitopes in the Sa and Cb sites [14]. Remarkably, many trimerization-dependent epitopes on HA persist after oligomer dissociation and fragmentation, demonstrating that complete folding of HA monomers requires trimerization [14].

Relative to the HA head, the HA stem exhibits little evolution among human IAV strains. This, and the failure to detect efficient neutralizing, HA stem-specific mAbs or polyclonal Abs (pAbs) [15], [16], led to a consensus that the stem region of HA is a poor vaccine target. Unfortunately, dogma-challenging findings from Okuno and colleagues clearly demonstrating the potential of StRAbs as broadly neutralizing agents [17], [18] were viewed as curiosities. All of this changed, however, when the isolation of mAbs from circulating B cells revealed that StRAbs are common in humans [19]–[24], and can be experimentally induced without difficulty in animals following a variety of immunization protocols [25]–[27].

These findings support the potential of HA stem-based immunogens as drift-resistant vaccines. Here, we examine the biogenesis of the principal site recognized by StRAbs. Our findings have important implications for immunogen design and possible viral escape from protective StRAbs.

## Results

### Biogenesis of HA Stem Epitopes Recognized by Mouse and Human mAbs

**Figure 1:**
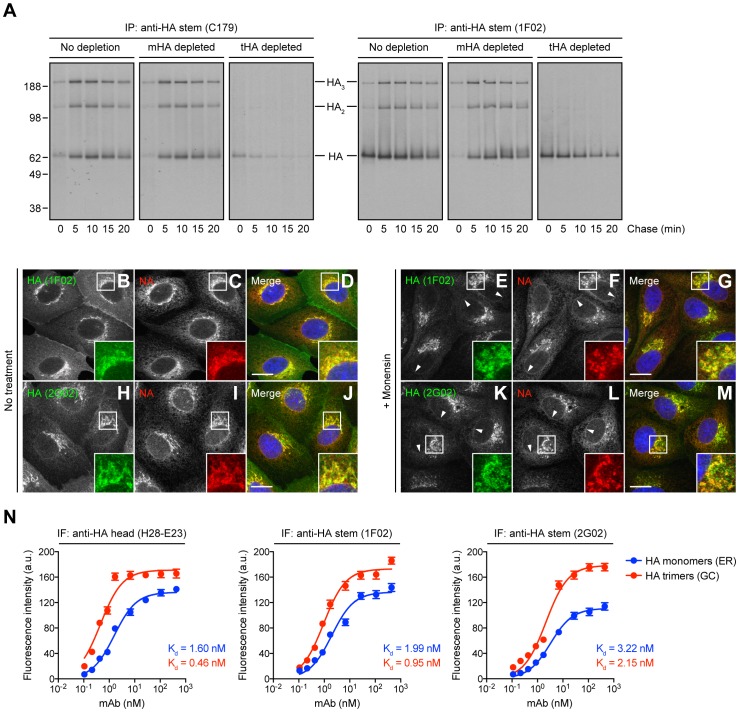
Identification of HA species recognized by StRAbs. (A) IAV PR8-infected MDCK cells were labeled with [^35^S]-Met and chased at 37°C. Detergent cell lysates were treated with an irrelevant mAb (10G-4 to the VSV N protein; no depletion) or depleted of HA monomers (mHA depleted) or HA trimers (tHA depleted) using the anti-HA head mAbs Y8-10C2 or H17-L2, respectively, at 4°C. Cell extracts were then incubated with the StRAbs C179 or 1F02 also at 4°C in a second round of immunoprecipitation (IP). Collected HA species were analyzed by non-reducing SDS-PAGE and fluorography. (B–M) MDCK cells were infected with IAV PR8 in the absence (no treatment) or presence of 10 µM monensin. Cells were fixed, permeabilized, and incubated with the human StRAbs 1F02 (B–G) and 2G02 (H–M) (green channel) and rabbit pAbs to NA (red channel). DNA was labeled using DAPI (blue channel). Stained cells were examined by fluorescence confocal microscopy. Bars: 10 µm. Arrowheads point NA co-localizing with HA monomers in the nuclear envelope (ER). (N) MDCK cells were infected with IAV PR8 in the presence of 10 µM monensin and processed for IF confocal microscopy using 2-fold serial dilutions of the purified anti-HA head mAb H28-E23 (control) or the StRAbs 1F02 and 2G02. Fluorescence intensities of the ER (HA monomers) and GC (HA trimers) are expressed as arbitrary units (a.u.). Data are represented as mean ± SEM from ∼100 cells/Ab dilution.

We examined the biogenesis of HA stem epitopes by 2 min radiolabeling IAV A/Puerto Rico/8/34 (PR8)-infected MDCK cells with [^35^S]-Met and chasing for up to 20 min at 37°C. We then recovered HA from non-ionic detergent (Triton X-100) lysates using mouse (C179 [18]) or human (1F02 [23]) StRAbs. We analyzed HA by sodium dodecyl sulfate-polyacrylamide gel electrophoresis (SDS-PAGE) under non-reducing conditions. Due to partial disulfide exchange post-lysis [14], oligomerized HA migrates as a mixture of denatured monomers, dimers, and trimers under these conditions (Fig. 1A). These StRAbs, like the HA monomer-specific, anti-HA head mAb Y8-10C2 [9], [14], bind to HA from the earliest chase time point, indicating their binding to monomeric HA (Fig. 1A). Moreover, C179 and 1F02 clearly recognize oligomeric forms of HA, as seen for the HA trimer-specific, anti-HA head mAb H17-L2 [9], [14] (Fig. 1A). Importantly, we observed similar reactivity to HA using other StRAbs that bind HAs from group 1 IAV, such as human 2A06, 2G02, and 3A01 [28] (Fig. S1). 2G02 is particularly interesting since it comprehensively neutralizes group 1 and group 2 IAV [28], [29], which suggests its probably binding to a different sight on the HA stem. It is worth mentioning, however, that StRAbs that bind to HA epitopes in a different manner than C179/CR6261 [30] might well behave differently. We also obtained similar results using a panel of drifted human IAV H1N1 strains, including the pandemic H1N1 2009 virus (Fig. S2).

To distinguish recognition of monomeric *vs.* trimerized HA, we used the HA monomer-specific mAb Y8-10C2 or the HA trimer-specific mAb H17-L2 to completely deplete cell lysates of HA monomers or trimers, respectively, prior to exposing lysates to either C179 or 1F02 [14]. This confirmed that C179 and 1F02 bind well to HA trimers, and that 1F02 also robustly binds HA monomers (Fig. 1A). C179 also clearly binds to HA monomers present in HA trimer-depleted samples, though less strongly than to HA trimers.

We extended these findings by examining the staining pattern of several StRAbs including human 2A06, 2G02, and 3A01, in addition to C179 and 1F02, on fixed and permeabilized MDCK cells 5 h post-IAV PR8 infection. Cells were infected in the presence of monensin, which greatly slows trafficking through the GC, to simplify the staining pattern, since we were essentially interested in the ability of the StRAbs to stain ER (HA monomers) *vs.* post-ER (HA trimers) compartments [14]. Cells were simultaneously stained with rabbit anti-IAV neuraminidase (NA) pAbs that localize NA throughout the entire secretory pathway. 1F02 (Fig. 1, B–G), 2G02 (Fig. 1, H–M), and other human StRAbs tested (Fig. S3) clearly recognize HA in both ER and GC, consistent with binding HA monomers and trimers, respectively. C179 (Fig. S3, A–F) binds trimerized HA in the GC and exhibits weak binding to HA monomers in the ER, consistent with the biochemical findings.

To further analyze the interaction of StRAbs with monomeric *vs.* trimerized HA, we developed a novel IF-based method to determine Ab avidity for HA in progressive stages of its biogenesis in IAV PR8-infected cells. This assay measures the Ab concentration required to give half-maximal staining of HA in various compartments by IF of fixed and permeabilized cells, exploiting the exclusive localization of HA monomers and trimers to ER *vs.* GC, respectively. We validated this method using H28-E23, a well-known anti-HA head mAb that binds monomeric and trimerized HA with similar avidity [9], [14] (Fig. 1N). Our data clearly demonstrate that human StRAbs also bind HA monomers and trimers with similar avidity (Fig. 1N). Note that the calculated avidity of all Abs examined is lower than the values obtained by ELISA (described below), what is probably related to the unavoidable effect of paraformaldehyde modification of Lys residues, commonly present on the HA surface.

Based on these findings, we conclude that StRAb epitopes are generated rapidly during the biogenesis of HA and are present on monomeric HA in a conformation that is highly similar to that present on trimerized HA.

### Processing of HA Glycans in the Distal Golgi Complex Impairs StRAb Binding

**Figure 2:**
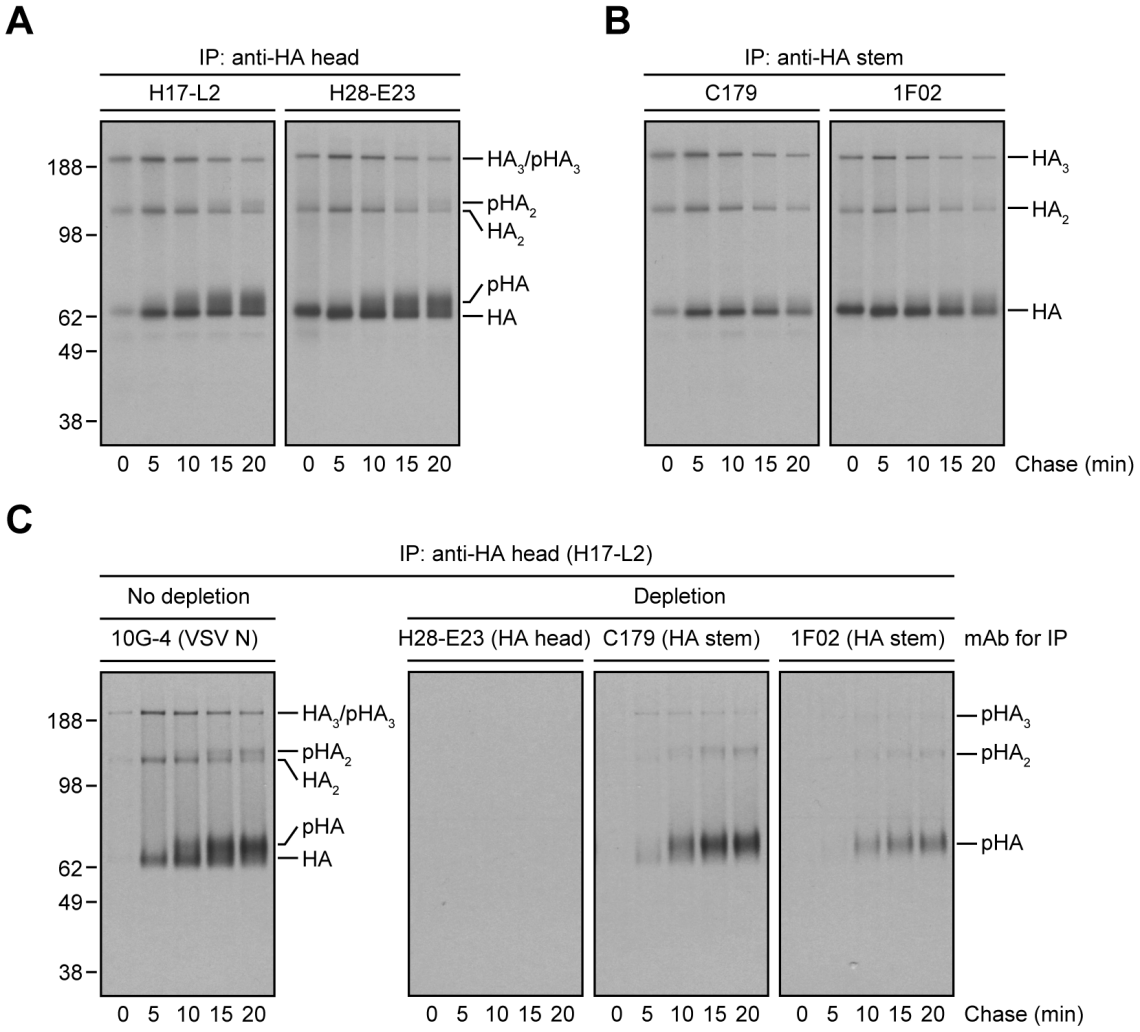
Processing of HA glycans in the distal Golgi complex shields HA from StRAb binding. (A and B) IAV PR8-infected MDCK cells were pulse-labeled with [^35^S]-Met and chased at 37°C. At the end of each chase time point cells were detergent-lysed and then subjected to IP with the anti-HA head mAbs H17-L2 and H28-E23 (A) or the StRAbs C179 and 1F02 (B) at 4°C. Immunocollected HA species were analyzed by non-reducing SDS-PAGE and fluorography. pHA: processed, glycosylated HA. (C) Detergent extracts from [^35^S]-Met-labeled and chased MDCK cells infected with IAV PR8 were treated with an irrelevant mAb (10G-4 to the VSV N protein; no depletion) or HA-depleted with the mAbs H28-E23 (control), C179, and 1F02 at 4°C as described in Fig. 1A before being incubated with H17-L2 also at 4°C in a second round of IP. Precipitated HA species were visualized by SDS-PAGE under non-reducing conditions and fluorography.

As previously observed [9], [14], [31], [^35^S]-Met pulse-labeled HA recovered by HA trimer- or HA monomer/trimer-specific mAbs (H17-L2 or H28-E23, respectively) demonstrates an initial slight increase in mobility in SDS-PAGE due to trimming of *N*-linked oligosaccharides in the early GC followed by a gradual decrease in mobility with further glycan modification in the distal GC (Fig. 2A). Remarkably, both mouse C179 and human 1F02 exhibited a greatly reduced ability to recover slowly migrating HA generated in the distal GC (Fig. 2B). Similar results were obtained using additional human StRAbs (2A06, 2G02, and 3A01; Fig. S1) or when examining 1F02 binding to HAs from a panel of drifted human IAV H1N1 strains (Fig. S2).

We extended this finding using StRAbs to deplete HA from [^35^S]-Met pulse-chased cell lysates. While H28-E23 completely depleted all HA species, as expected, 1F02 and particularly C179 failed to remove HA with mature *N*-linked glycans (Fig. 2C).

**Figure 3:**
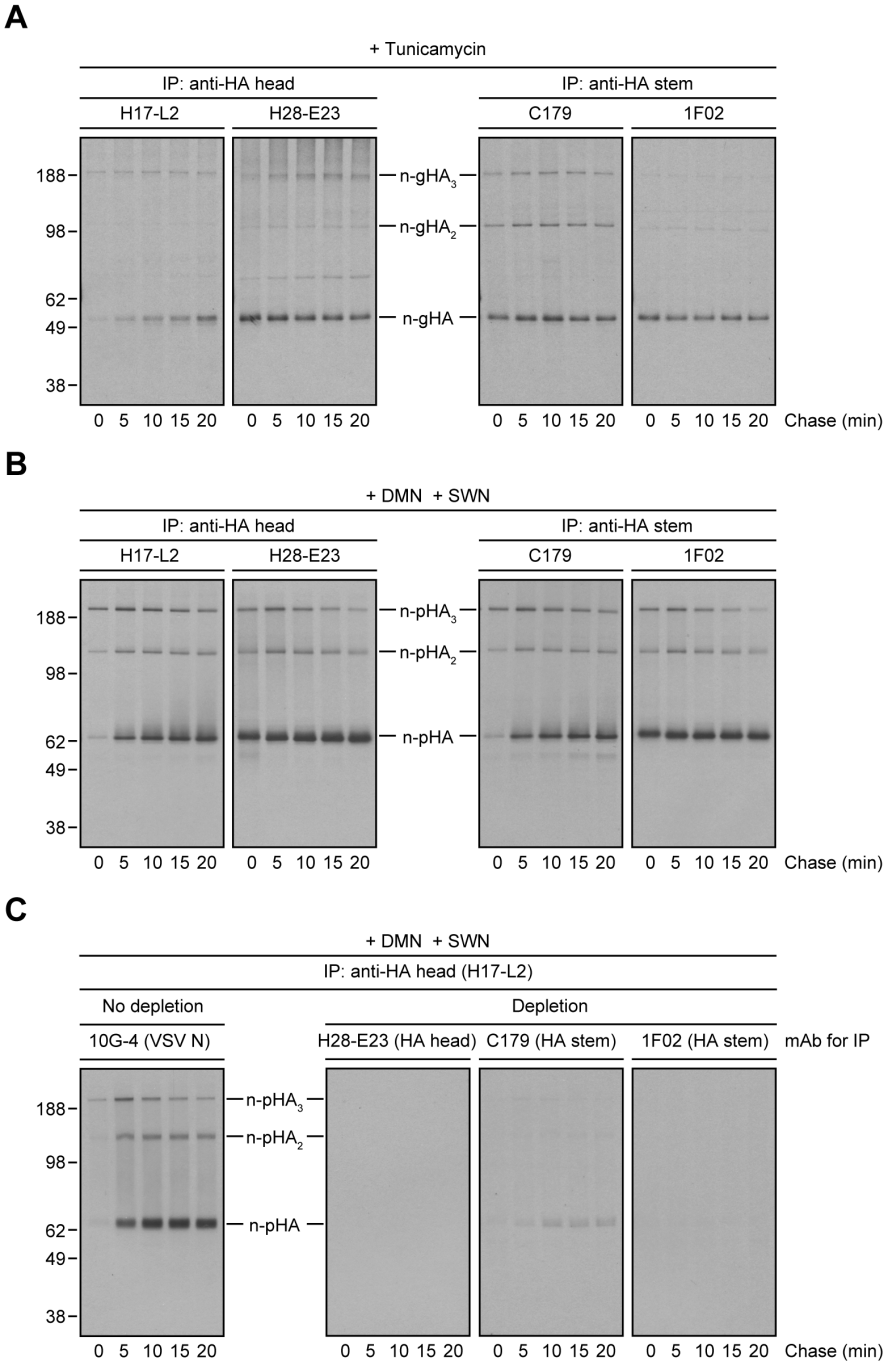
Inhibition of *N*-linked glycosylation or *N*-linked oligosaccharide processing restores proper StRAb binding to HA. (A–C) MDCK cells infected with IAV PR8 were treated with tunicamycin (A) or a mixture of DMN and SWN (B and C) for 30 min before being labeled with [^35^S]-Met and chased in continuous presence of the inhibitors at 37°C. (A and B) Detergent cell extracts were subjected to IP using the anti-HA head mAbs H17-L2 and H28-E23 or the StRAbs C179 and 1F02 at 4°C. Immunocollected proteins were resolved by non-reducing SDS-PAGE and visualized by fluorography. n-gHA: non-glycosylated HA; n-pHA: non-processed, glycosylated HA. (C) Detergent cell lysates were incubated with an irrelevant mAb (10G-4 to the VSV N protein; no depletion) or HA-depleted with the mAbs H28-E23 (control), C179, and 1F02 at 4°C before being treated with H17-L2 also at 4°C in a second round of IP as described in Fig. 2C. Precipitated HA species were analyzed by non-reducing SDS-PAGE and fluorography.

If the loss of StRAb binding is due to HA *N*-linked oligosaccharide modifications in the distal GC, preventing *N*-linked glycosylation or blocking *N*-linked glycan processing should enable StRAbs to bind HA throughout a long chase. Treatment of IAV PR8-infected cells with tunicamycin to prevent *N*-linked glycosylation (Fig. 3A) or a mixture of 1-deoxymannojirimycin (DMN) and swainsonine (SWN) to inhibit Golgi α-mannosidases I and II, respectively (Fig. 3B), completely blocked the mobility shift associated with *N*-linked oligosaccharide processing and enabled C179 and 1F02 (Fig. 3, A and B) as well as 2A06, 2G02, and 3A01 (Fig. S4) to maintain binding to HA 20 min post-[^35^S]-Met pulse. In line with the experiments described above, StRAbs nearly (C179) or fully (1F02) depleted all forms of HA present in radioactive lysates from cells treated with Golgi α-mannosidases inhibitors (Fig. 3C; compare with Fig. 2C).

**Figure 4:**
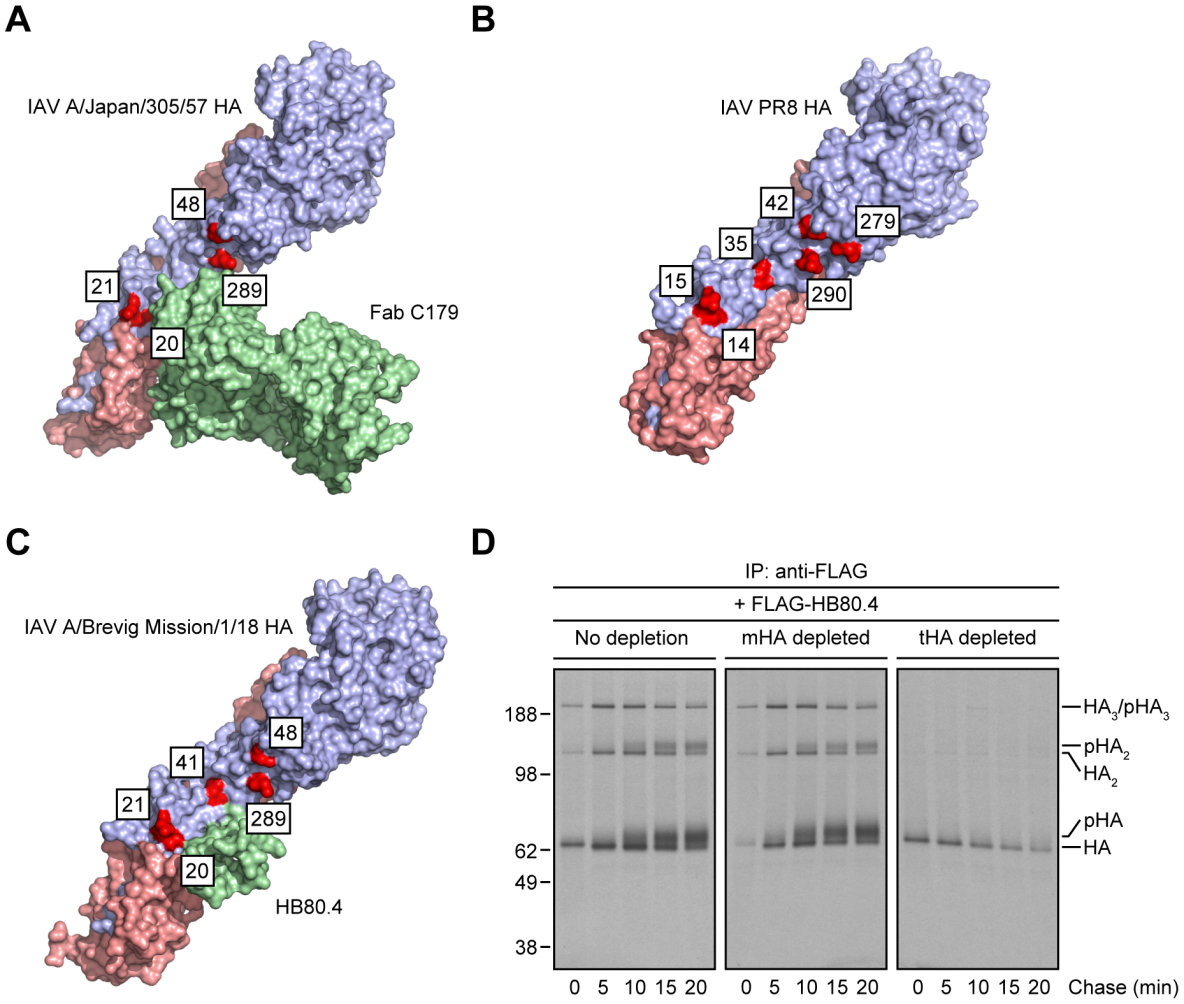
Steric hindrance due to *N*-linked glycan processing shields HA from StRAb binding. (A–C) PyMOL images of the crystal structures of mouse Fab C179 in complex with the IAV A/Japan/305/57 (H2N2) HA monomer [RSCB protein database entry: 4HLZ) [32] (A)]; the IAV PR8 HA monomer used in this study [shown for comparison only; RSCB protein database entry: 1RVX) [35] (B)]; and HB80.4 in complex with the IAV A/Brevig Mission/1/18 (H1N1) HA monomer [RSCB protein database entry: 4EEF) [36] (C)], showing glycosylation-prone Asn residues within or around of the stem region of HA (red; H3 numbering scheme). The HA1 and HA2 peptides are displayed in purple and pink, respectively. (D) Detergent extracts from [^35^S]-Met-labeled and chased IAV PR8-infected MDCK cells at 37°C were treated with an irrelevant mAb (10G-4 to the VSV N protein; no depletion) or depleted of HA monomers (mHA depleted) or HA trimers (tHA depleted) using the anti-HA head mAbs Y8-10C2 or H17-L2, respectively, at 4°C as described in Fig. 1A. Detergent cell lysates were then incubated with 2.4 µg/ml FLAG-tagged HB80.4 also at 4°C. HA species in complex with HB80.4 were immunocollected with the anti-FLAG mAb M2 at 4°C and analyzed by non-reducing SDS-PAGE and fluorography. pHA: processed, glycosylated HA.

Crystal structures of StRAbs bound to HA reveal that *N*-linked oligosaccharides tightly ring the Ab (Fig. 4A shows mouse Fab C179 binding to an H2 HA [32]; human StRAbs bind HA similarly [21], [30], [33], [34]; Fig. 4B shows the IAV PR8 HA used in this study for comparison only [35]), consistent with the idea that addition of saccharide subunits in the GC increases the steric hindrance sufficiently to decrease the StRAb binding avidity. This predicts that smaller ligands will not be affected by GC *N*-linked oligosaccharide modifications. We confirmed this prediction using HB80.4, a tiny (65 residues), computationally designed protein that binds to the same region of the HA stem as StRAbs with nM avidity (HB80.4 binding to an H1 HA is shown in Fig. 4C [36]). Unlike the StRAbs used in this study, HB80.4 bound all HA species in radioactive pulse-chase experiments (Fig. 4D), including HAs from a panel of drifted human IAV H1N1 strains (Fig. S5).

All together, these findings demonstrate first, that StRAbs bind to the HA stem independently of *N*-linked glycosylation and second, that modification of HA glycans, presumably those located in the proximity of the StRAb epitopes, interferes with StRAb binding to HA under the conditions we have employed.

### StRAb Binding to Virus-Derived HA Is Temperature-Sensitive

**Figure 5:**
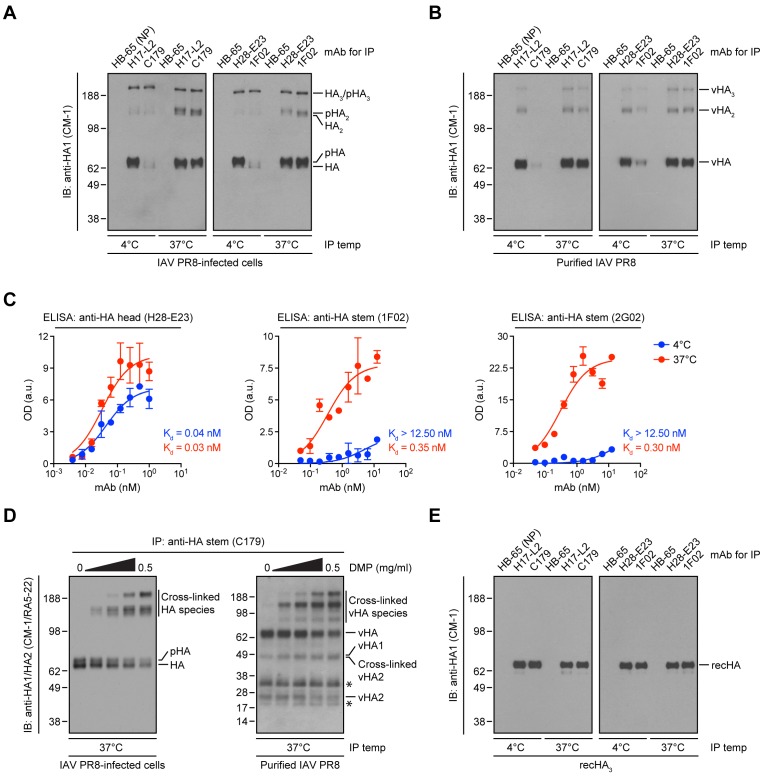
Temperature-dependent StRAb binding to cell-derived mature HA or viral HA. (A and B) IAV PR8-infected MDCK cells (A) or purified IAV PR8 (B) were detergent-lysed and incubated with an irrelevant mAb (HB-65 to the IAV NP protein; negative control), the anti-HA head mAbs H17-L2 and H28-E23, or the StRAbs C179 and 1F02 at 4°C or 37°C. Precipitated HA species were resolved by non-reducing SDS-PAGE and visualized by IB using the denatured HA-specific, anti-HA1 mAb CM-1. pHA: processed, glycosylated HA; vHA: viral HA. (C) ELISA plates coated with egg-grown IAV PR8 were treated with the mouse anti-HA head mAb H28-E23 (control) or the human StRAbs 1F02 and 2G02 at 4°C or 37°C, followed by primary Ab detection using HRP-conjugated anti-mouse or anti-human Abs, respectively. Optical density (OD) values are expressed as arbitrary units (a.u.). Data are represented as mean ± SEM from one experiment done in duplicate and repeated at least three times with similar results. (D) Detergent cell or viral lysates were cross-linked with increasing amounts of DMP. After addition of an excess of Tris-HCl to quench the reaction, cross-linked lysates were incubated with the StRAb C179 at 37°C. Immunocollected proteins were analyzed by SDS-PAGE under reducing conditions and IB using CM-1 and the denatured HA-specific, anti-HA2 mAb RA5-22. Asterisk: non-specific band. (E) Recombinant HA trimers (IAV PR8-foldon-His_6_ recHA; recHA_3_) purified from insect cells were resuspended in 1% Triton X-100 and subjected to 4°C or 37°C IP as described in A and B. Precipitated recHA_3_ was visualized by non-reducing SDS-PAGE and IB using CM-1.

Our results predict that StRAbs should bind poorly to virion HA, due to the presence of mature *N*-linked oligosaccharides. To test this, we purified IAV PR8 from MDCK cell supernatants, collected HA from Triton X-100 lysates with anti-HA head (H17-L2 and H28-E23) or anti-HA stem (C179 and 1F02) mAbs, and measured bound HA by immunoblotting (IB) using the mAb CM-1 (specific for a linear epitope in HA1) [14]. While H17-L2 and H28-E23 efficiently recovered mature (*N*-linked glycan-processed) HA from IAV PR8-infected cell lysates (Fig. 5A) and viral HA (Fig. 5B), neither C179 nor 1F02 detectably bound cell-derived mature HA (Fig. 5A) or viral HA (Fig. 5B) under these conditions. In line with our previous observations, StRAbs only reacted with non-processed HA species present in detergent lysates made from cells infected with IAV PR8 (Fig. 5A).

These findings are at odds with the biological activity exerted by C179, 1F02, and other StRAbs in virus neutralization and protection experiments. Until this point, in all of the experiments shown, we incubated Abs with HA at 4°C, a precaution typical for biochemical work (low temperature limits the activity of proteases and other enzymes). Abs do not physiologically function at 4°C: neutralization assays *in vitro* are typically performed at 37°C, and *in vivo* means 37°C in non-febrile humans, and several degrees higher in mice, birds, and febrile patients.

Indeed, we previously showed that Y8-10C2, a mAb absolutely specific to HA monomers at 4°C, binds HA trimers at 37°C due to the increased flexibility of the HA globular domains that enable Ab access to its epitope at the trimer interface [37]. Could a similar phenomenon apply to StRAbs? Incubation of StRAbs with IAV PR8-infected cell lysates or detergent lysates from purified virus at 37°C resulted in the recovery of similar amounts of cell-derived mature HA (Fig. 5A) or viral HA (Fig. 5B) as observed with HA head-specific mAbs. We further observed temperature-dependent binding of the human StRAbs 1F02 and 2G02 in ELISA using whole IAV PR8 as antigen, while the anti-HA head mAb H28-E23 bound IAV PR8 in a temperature-independent manner, as expected (Fig. 5C).

The accessibility of the StRAb epitope at 37°C might be related to the mobility of the oligomerized HA stem domains relative to each other, as required for the binding of Y8-10C2 to its epitope on trimerized HA [37]. To assess this possibility, we cross-linked HA present in detergent extracts from IAV PR8-infected cells or purified virus with the non-cleavable cross-linker dimethyl pimelimidate (DMP) before being incubated at 37°C with the mouse StRAb C179. We analyzed bound HA via reducing SDS-PAGE and IB using a mixture of CM-1 and the denatured HA-specific, anti-HA2 mAb RA5-22 [14]. Under these conditions, HA oligomers completely dissociate and unfold [14]. However, DMP-mediated cross-linking of HA reduced its dissociation in a concentration-dependent manner as seen by decreasing amount of HA monomers and increasing amounts of HA oligomers (Fig. 5D). Importantly, with increasing concentrations of cross-linker the amount of C179-reactive HA remains relatively constant (Fig. 5D). This strongly implies that the temperature-dependent reactivity of StRAbs to HA relies on the enhanced flexibility of complex *N*-linked glycans rather than the HA polypeptide itself.

Taken together, these findings demonstrate that StRAb binding to HA exhibits a unique sensitivity to *N*-linked oligosaccharide structures that is highly dependent on temperature.

### Recombinant HA: When a Liability Becomes an Asset

The ability to generate large amounts of native HA from cultured insect cells is a boon for both basic science and vaccine development. One of the theoretical limitations of this system is the limited capacity of insect cells to generate complex *N*-linked glycans. Our findings predict, however, that the failure to modify simple *N*-linked oligosaccharides will enhance, and not limit HA stem antigenicity/immunogenicity.

Indeed, using HA trimers purified from insect cells [38] in a highly native state [14] we found that binding of StRAbs occurs perfectly well at 4°C (Fig. 5E), as measured under the same conditions used in Fig. 5, A and B. Thus, antigenicity of the HA stem is fortuitously enhanced by a failure of the producer cell to faithfully recapitulate the biogenesis of HA *in vivo*.

## Discussion

Although many questions remain to be answered, there is a tremendous enthusiasm regarding the potential of StRAbs to improve IAV vaccination. At the very least, StRAbs should be useful therapeutically in severe influenza. At the very most, vaccines that effectively induce StRAbs could provide long lasting protection and greatly diminish the annual toll of seasonal IAV and prevent pandemics arising from introduction of novel HA subtypes into the human population from the enormous animal reservoir [39], [40].

Effectively inducing StRAbs requires more detailed knowledge of the B cell response to HA. It is generally believed that the greater intrinsic immunogenicity of the HA globular domain suppresses Ab responses to the HA stem. This remains to be established experimentally, however, and it will be of interest whether this is due strictly to an enhanced ability of anti-HA head B cells to compete for limiting amounts of HA in primary and secondary immune responses. If this is the case, then there are several potential strategies to boost HA stem immunogenicity.

First, the immunogenicity of the HA globular domain can potentially be minimized by engineering HA to contain a maximal number of *N*-linked oligosaccharides to shield the antigenic regions. The H1 and H3 HAs demonstrated a clear tendency to increase the numbers of *N*-linked glycans in the head [41], [42], suggesting that this might be possible. In line with this hypothesis, Eggink and colleagues recently designed an HA with a hyper-glycosylated globular domain that greatly increases the mouse broadly neutralizing StRAb response [43].

Second, vaccines could be formulated with high avidity Abs (natural or particularly designed Abs like HB80.4) specific for the HA head to block activation of B cells specific for this region. A natural, much cheaper, and probably more effective alternative method is to simply immunize individuals with vaccines to prior IAV strains to which they already possess high anti-HA globular domain Ab titers. A related strategy is to exploit the original antigenic sin [44], *i.e.* the ability of secondary B cells to immunodominate primary B cells, and immunize with vaccines containing HA with a completely novel head and a conserved stem region [45]–[47]. This requires, however, that individuals already possess a sufficient number of secondary B cells making StRAbs, so it cannot be used for children receiving their first IAV vaccine. Indeed, it may be critical to focus on truly naïve individuals, since true to its name, original antigenic sin may increase the difficulty of inducing effective StRAb responses in individuals with immunodominant Ab responses specific for the globular domain.

Third, the stem region of HA alone could be used as a vaccine immunogen. Protective StRAbs are highly dependent on HA conformation, as indicated by their inability to bind acid-triggered HA or denatured HA in IBs [30]. Generating folded HA stem-only immunogens could be difficult, particularly if StRAb binding requires HA oligomerization, as concluded by Krammer and colleagues [48] studying soluble HA produced by insect cells. By contrast, using a novel, widely applicable IF-based method to measure Ab avidity for antigens *in situ* (in this case, fixed and permeabilized IAV-infected cells), we found that all human StRAbs we tested bind HA monomers with nM avidity (although in the case of mouse C179 with clearly diminished avidity) (Figs. 1, S1, S2, and S3). Krammer *et al.*'s failure to detect StRAb binding to monomeric HA could be related to the absence of the COOH-terminal residues in the recombinant HAs used [48], but in any event, it shows the importance of carefully characterizing the antigenicity of recombinant vaccine constructs. Our findings support the concept of inducing StRAbs with recombinant HA monomers, obviating the need for complex trimerization/foldon domains. While the engineering of immunogenic HA stem-only immunogens is challenging [26], Bommakanti and colleagues [49], [50] describe rationally designed trimerized HA stem-only constructs that bound and induced neutralizing StRAbs. In deploying such vaccines it is essential to be cognizant of the possible deleterious effects of immunogens that *enhance* pathogenicity, as recently reported by Khurana *et al.* [51]. Though this phenomenon to date is limited to the swine model [51], a similar phenomenon might account for Ab-enhanced human disease with recently shifted viruses [52].

One of our most interesting findings is the temperature dependence of StRAb binding to mature HA (Fig. 5). Mammals evolved to carefully control their temperatures, and Ab binding at 4°C is clearly irrelevant to StRAb function *in vivo*. This is not, however, germane to the major conclusions of our study. Rather, our findings provide novel mechanistic details regarding the participation of *N*-linked oligosaccharides in the thermodynamics of the StRAb-HA interaction. To our knowledge, this is only the second example of temperature-dependent neutralizing Ab binding to IAV HA, the first being our 1993 study where we reported the temperature-dependent binding of Y8-10C2, a mAb specific for the HA Sa antigenic site [37], to HA trimers. Y8-10C2 temperature dependence is based on the “breathing” of the HA globular domains to enable Ab access to its epitope, which is sterically blocked by the close proximity of neighboring monomers in trimerized HA in its minimal energy state. The effect of the temperature-dependent conformational breathing on binding of neutralizing Abs to viruses is now a topic of great interest and immediately relevant to HIV-1 and flavivirus vaccine development [53], [54].

The temperature dependence of StRAb binding to HA is likely due not to the conformational gyrations of the HA polypeptide *per se*, but rather to the flexibility of the *N*-linked oligosaccharides that surround the StRAb-binding region, since chemical cross-linking of HA trimers to limit polypeptide flexibility does not alter the temperature-dependent StRAb binding to HA (Fig. 5). Thus, HB80.4, a small-engineered protein ligand with a very similar binding footprint, binds in a temperature-independent manner (Figs. 4 and S5). This observation demonstrates the potential superiority of tiny, computationally designed Abs that are not subject to glycan mediated interference, almost certainly due to their small size. Most directly, StRAb-temperature dependence is abrogated by either preventing HA glycosylation with tunicamycin or HA glycan maturation with Golgi α-mannosidases inhibitors in mammalian cells (Figs. 3 and S4), or by expressing HA in insect cells (Fig. 5), which typically do not generate complex *N*-linked oligosaccharides [55].

Wang and colleagues [56] and more recently Chen *et al.* [57] engineered a recombinant HA carrying simple sugars at each glycosylation site that, used as vaccine, elicits enhanced cross-strain neutralization and protection in mice and ferrets. Thus, our findings have also important practical impact, since they provide basic information that explains the improved ability of HA with simple *N*-linked oligosaccharides to elicit StRAbs and provide broader immunity to drifted and shifted HAs [56], [57], what is consistent with a temperature-dependent glycan steric hindrance of StRAb binding to mature and viral HA.

If StRAb binding to HA is so precarious, could effective large-scale HA stem vaccination in humans generate sufficient immune pressure to arise HA stem escape variants? Given the high conservation of *N*-linked oligosaccharide sites in the stem region of HA [41], the fitness costs of adding a new site in the StRAb footprint are likely to be sufficiently severe to preclude this escape strategy. On the other hand, amino acid substitutions in or around of the HA stem could re-orient the existing *N*-linked glycans to increase their steric blockade effect, since examination of the crystal structures of StRAbs bound to HA reveals a close tolerance between the Ab and several *N*-linked oligosaccharides located in the HA stem (Fig. 4). Reported difficulties in generating StRAb-escape mutants *in vitro* [21], [27], [30] strongly suggests that viral escape requires changing multiple residues, either to attain escape itself or to regain fitness lost from altering the amino acid(s) required for escape. Compensatory mutations could be also located at a considerable distance from the HA stem epitope, as shown by Wang and colleagues [58]. In this unique example of StRAb viral escape to date, a point mutation on an IAV H1N1 HA, L415I, distant from any known StRAb antigenic site, presumably induces a conformational change on the HA stem domain in order to fit the bulkier side chain of Ile [58]. Likewise, the isolation of Y8-10C2 escape mutants revealed that the stem region of HA controls the Ab accessibility to its epitope in the Sa site at the HA trimer interface [37].

Although viral escape from StRAbs is likely to be difficult, our findings suggest that given enough time and immune pressure, escape via a conformational mechanism is a real possibility. These are not necessarily unmitigated bad news, however, since mutations may compromise the HA function sufficiently to reduce viral pathogenicity or transmissibility.

## Materials and Methods

### Cells

MDCK cells (American Type Culture Collection; Manassas, VA) were maintained in complete medium (Dulbecco's modified Eagle's medium+GlutaMAX-I (Gibco; Grand Island, NY), supplemented with 4.5 mg/ml D-glucose, 110 µg/ml sodium pyruvate, and 7.5% heat-inactivated fetal bovine serum) at 37°C in a humidified atmosphere of 9% carbon dioxide in air.

### Virus and Viral Infections

IAV PR8 was grown in the allantoic cavity of embryonated chicken eggs and conserved as infectious allantoic fluid at −80°C. MDCK monolayers were washed twice with Dulbecco's phosphate-buffered saline (DPBS; Gibco) and incubated with AIM medium (Minimum essential medium+GlutaMAX-I, supplemented with Earle's salts, 0.1% bovine serum albumin, and 20 mM Hepes pH 6.6) containing 10 infectious doses per cell of egg-grown IAV PR8 at 37°C in a humidified atmosphere of 9% carbon dioxide in air. After 1 h, the infection medium was replaced by complete medium and incubation continued for additional 4 h. Infection of MDCK cells with IAV A/Fort Monmouth/1/47, A/Denver/1/57, A/New Caledonia/20/99, or A/California/7/09 was carried out for 17 h under the same culture conditions.

### Purification of IAV PR8 from Mammalian Cell Supernatants

Confluent monolayers of MDCK cells growing in 175 cm^2^ culture flasks (Thermo Scientific Nunc; Rochester, NY) were extensively washed with DPBS and infected with 1 infectious dose per cell of egg-grown IAV PR8 in TCID50 medium (Minimum essential medium+GlutaMAX-I, supplemented with 1 mM Hepes pH 7.5, 1 µg/ml trypsin TPCK-treated (Worthington; Lakewood, NJ), and 50 µg/ml gentamycin) for three days at 37°C in a humidified atmosphere of 9% carbon dioxide in air. Cell supernatants were layered on the top of a 20% (w/v) sucrose cushion and ultracentrifuged in a SW38 rotor (Beckman Coulter Inc.; Fullerton, CA) for 4 h at 24,000× *g*, 4°C. The viral pellet was resuspended in DPBS supplemented with calcium and magnesium (DPBS-Ca/Mg; Gibco), re-layered the on top of a 15–60% (w/v) sucrose gradient, and ultracentrifuged in a SW41 rotor (Beckman Coulter Inc.) for 2 h at 35,000× *g*, 4°C. Purified IAV PR8 were collected from between the 15% and 60% sucrose interface, ultracentrifuged again to clear residual sucrose, and resuspended in DPBS-Ca/Mg. Viral stocks were stored at 4°C.

### Antibodies

The mouse StRAb C179 was purchased from Takara Bio, Inc.-Clontech (Mountain View, CA). All human StRAbs used in this study were described in previous publications [23], [28]. Mouse mAbs Y8-10C2, H17-L2, and H28-E23 to the HA globular domain, HB-65 to the NP protein, and 10G-4 to the VSV N protein, as well as rabbit anti-NA pAbs were reported elsewhere [9], [59]–[61]. FLAG-tagged HB80.4 was described previously [36]. The mouse anti-FLAG mAb M2 was from Sigma-Aldrich (Saint Louis, MO). DyLight 488-conjugated donkey anti-mouse IgG, Alexa Fluor 488-conjugated donkey anti-human IgG, and DyLight 546-conjugated donkey anti-rabbit IgG were from Jackson ImmunoResearch Laboratories, Inc. (West Grove, PA). The HRP-conjugated anti-mouse IgG TrueBlot ULTRA was obtained from eBioscience (San Diego, CA).

### Radioactive Pulse-Chase Analysis, Immunoprecipitation, and Immunoblotting

Equivalent amounts of IAV PR8-infected MDCK cells were pulse-labeled for 2 min with [^35^S]-Met (PerkinElmer; Waltham, MA) at 37°C, chased at the same temperature, detergent lysed, and subjected to IP as described [14]. When indicated, tunicamycin (5 µg/ml) or a mixture of DMN (1 mM) and SWN (10 µM) (Calbiochem; Billerica, MA) were added to cells 30 min before radiolabeling and maintained throughout the radioactive pulse and chase periods. Immunocollected proteins were analyzed by SDS-PAGE and visualized by exposing dried gels to Carestream Kodak BioMax MR (Sigma-Aldrich) films. Gels were stained with Coomassie brilliant blue R (MP Biomedicals, LLC; Santa Ana, CA) prior to drying to ensure that the Abs were equally recovered and loaded in all lanes. Since equal amounts of IgG were added to each sample, this served as a loading control. IB studies were carried out as reported [62].

### Immunofluorescence Confocal Microscopy

MDCK cells growing on cover glasses (Marienfeld GmbH & Co.KG; Lauda-Königshofen, Germany) were infected with IAV PR8 in the absence or presence of 10 µM monensin (Sigma-Aldrich) and prepared for IF confocal microscopy as described previously [14]. Inverted coverslips were mounted on microscope slides using Fluoromount G with DAPI (Electron Microscopy Sciences; Hatfield, PA) and examined with a TCS SP5 (DMI 6000) confocal microscope system (Leica Microsystems; Deerfield, IL).

### Antibody Binding Analysis by Immunofluorescence Confocal Microscopy

MDCK cells were infected with IAV PR8 in the presence of 10 µM monensin and processed for IF confocal microscopy using 2-fold serial dilutions of purified mAbs (from 500 nM to 0.05 nM). ∼100 cells/Ab dilution were imaged using the same microscope and image acquisition settings. Fluorescence intensities of HA monomers in the ER and HA trimers in the GC were measured on background-subtracted images using the ImageJ software (http://rsbweb.nih.gov/ij). Ab K_d_ values were then calculated with the Prism 6 software (GraphPad; La Jolla, CA) by fitting the hyperbola directly to the saturation isotherm by non-linear regression.

### Chemical Cross-linking

IAV PR8-infected MDCK cells or purified IAV PR8 were washed twice with DPBS and lysed with ice-cold lysis buffer (0.5% Triton X-100, 200 mM triethanolamine pH 8, 150 mM NaCl, and 5 mM EDTA) supplemented with the complete, Mini, EDTA-free protease inhibitor cocktail (Roche Diagnostics, Indianapolis, IN). Equivalent amounts of detergent lysates were incubated with increasing amounts (0, 0.05, 0.1, 0.25, and 0.5 mg/ml) of the cross-linker DMP (Thermo Scientific Pierce; Rockford, IL) for 3 h at room temperature. The reaction was stopped by adding 50 mM Tris-HCl pH 7.5 to each sample.

### ELISA

96-well ELISA plates (Greiner Bio-One; Monroe, NC) were prepared by adding 100,000 TCID50 egg-grown IAV PR8 diluted in DPBS to each well. After overnight incubation at 4°C, plates were washed three times with DPBS+0.05% Tween-20, and pre-cooled on ice prior to Ab addition. Abs were incubated on plates for 2 h at 4°C or 37°C. After extensively washing with DPBS+0.05% Tween-20, bound primary Abs were detected by incubating plates with HRP-conjugated rat anti-mouse IgG kappa light chain (H28-E23) or goat anti-human IgG kappa light chain (1F02 and 2G02) (SouthernBiotech; Birmingham, Al) for 1 h at 4°C. ELISA plates were washed again with DPBS+0.05% Tween-20 and incubated with the SureBlue TMB Microwell Peroxidase Substrate (KPL; Gaithersburg, MD) for 5 min at room temperature. The reaction was stopped by adding 1 M HCl to each sample. The ODs were determined at 450 nm on a Spectra Max M5 microplate reader (Molecular Devices; Downington, PA) and the Ab K_d_ values were then calculated using the Prism 6 software.

## Supporting Information

Figure S1 **Binding of additional human StRAbs to monomeric HA and non-processed HA trimers.** IAV PR8-infected MDCK cells were labeled with [^35^S]-Met and chased at 37°C. Detergent cell extracts were incubated with the HA monomer/trimer-specific, anti-HA head mAb H28-E23 (control) or the StRAbs 2A06, 2G02, and 3A01 at 4°C. Immunocollected HA species were visualized by non-reducing SDS-PAGE and fluorography. pHA: processed, glycosylated HA.
(TIF)

Figure S2 **StRAbs recognize HA monomers and non-processed, trimerized HA from additional drifted human IAV H1N1 strains.** MDCK cells infected with IAV A/Fort Monmouth/1/47, A/Denver/1/57, A/New Caledonia/20/99, or A/California/7/09 were [^35^S]-Met pulse-labeled and chased at 37°C before being detergent-lysed. HA species were precipitated with the StRAb 1F02 at 4°C and then analyzed by non-reducing SDS-PAGE and fluorography.
(TIF)

Figure S3 **Reactivity of additional StRAbs to HA monomers and trimers assayed by immunofluorescence confocal microscopy.** (A–R) MDCK cells were infected with IAV PR8 in the absence (no treatment) or presence of 10 µM monensin as described in Fig. 1, B–M. HA was labeled on fixed and permeabilized cells using the mouse StRAb C179 (A–F) or the human StRAbs 2A06 (G–L) and 3A01 (M–R) (green channel). NA was detected using rabbit pAbs (red channel). DNA was visualized using DAPI (blue channel). Stained cells were examined by fluorescence confocal microscopy. Bars: 10 µm. Arrowheads point NA co-localizing with HA monomers in the nuclear envelope (ER).
(TIF)

Figure S4 **Inhibition of** ***N*** **-linked glycan processing allows proper StRAb binding to HA.** IAV PR8-infected MDCK cells were treated with a mixture of DMN and SWN before being pulse-labeled with [^35^S]-Met and chased at 37°C in the continuous presence of the inhibitors as described in Fig. 3, B and C. Detergent cell extracts were incubated with the anti-HA head mAb H28-E23 (control) or the StRAbs 2A06, 2G02, and 3A01 at 4°C. Immunocollected proteins were resolved by SDS-PAGE under non-reducing conditions and visualized by fluorography. n-pHA: non-processed, glycosylated HA.
(TIF)

Figure S5 **HB80.4 overcomes** ***N*** **-linked oligosaccharide shielding within the stem region of HAs from additional drifted human IAV H1N1 strains.** MDCK cells infected with IAV A/Fort Monmouth/1/47, A/Denver/1/57, A/New Caledonia/20/99, or A/California/7/09 were labeled with [^35^S]-Met and chased at 37°C. Detergent cell lysates were incubated with FLAG-tagged HB80.4 at 4°C. HA species in complex with HB80.4 were precipitated with the anti-FLAG mAb M2 also at 4°C and then analyzed by non-reducing SDS-PAGE and fluorography. Asterisk: non-specific band; pHA: processed, glycosylated HA.
(TIF)
