# Supplementary figures and images for: Biogenesis of Influenza A Virus Hemagglutinin Cross-Protective Stem Epitopes
Source: PLoS Pathog. 2014 Jun 12;10(6):e1004204. doi: 10.1371/journal.ppat.1004204 (PMC4055778; doi:10.1371/journal.ppat.1004204)

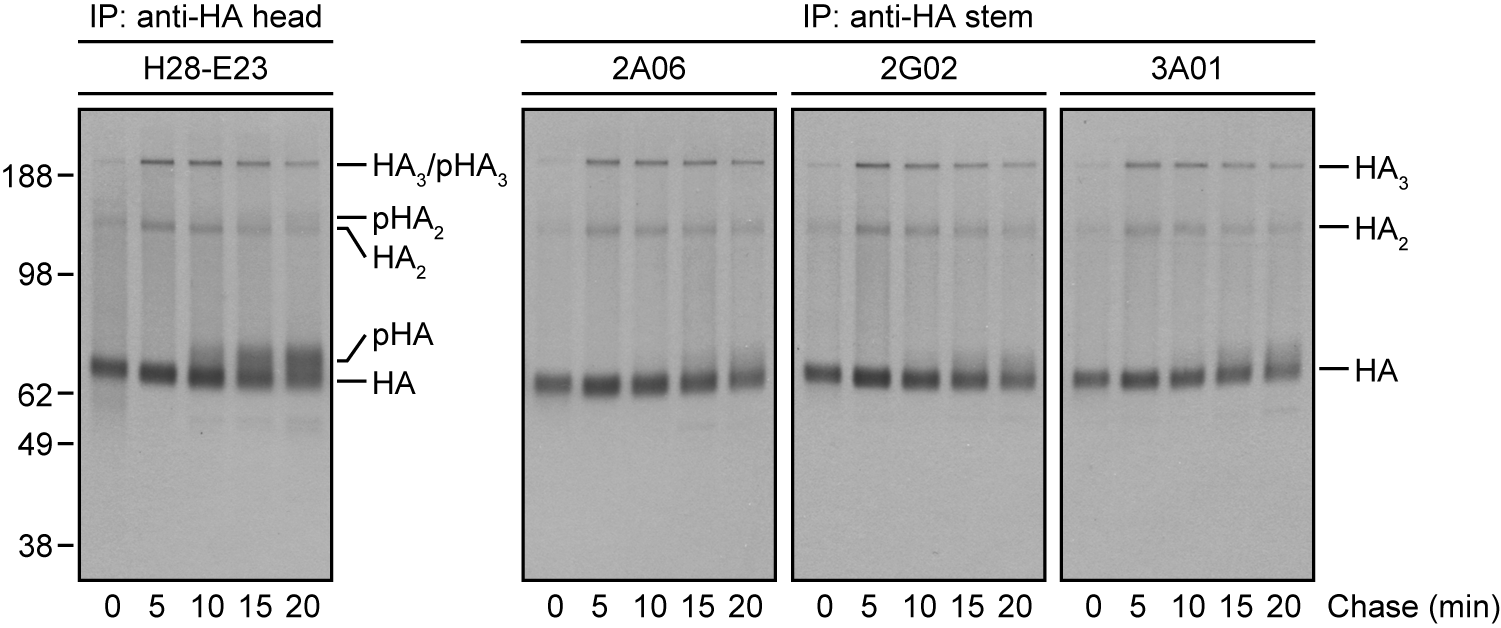

Supplement: Figure S1 — Binding of additional human StRAbs to monomeric HA and non-processed HA trimers. IAV PR8-infected MDCK cells were labeled with [35S]-Met and chased at 37°C. Detergent cell extracts were incubated with the HA monomer/trimer-specific, anti-HA head mAb H28-E23 (control) or the StRAbs 2A06, 2G02, and 3A01 at 4°C. Immunocollected HA species were visualized by non-reducing SDS-PAGE and fluorography. pHA: processed, glycosylated HA. (TIF) [file ppat.1004204.s001.tif]

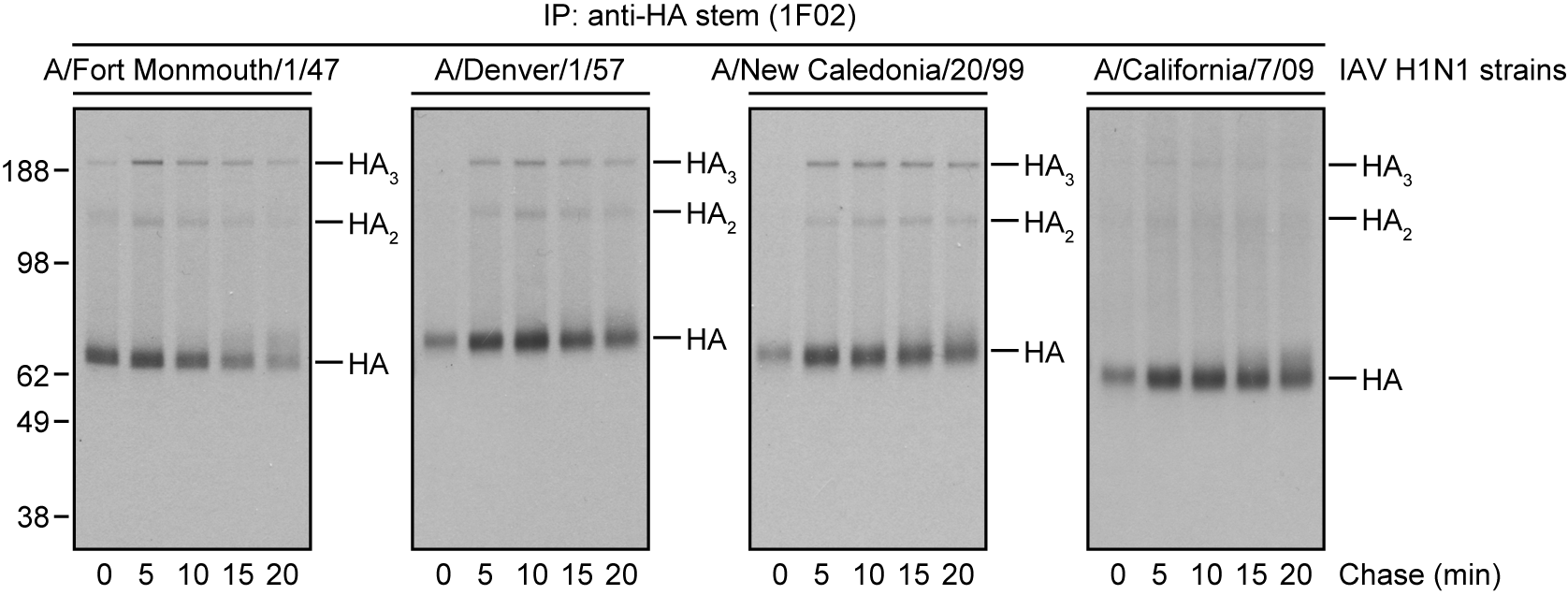

Supplement: Figure S2 — StRAbs recognize HA monomers and non-processed, trimerized HA from additional drifted human IAV H1N1 strains. MDCK cells infected with IAV A/Fort Monmouth/1/47, A/Denver/1/57, A/New Caledonia/20/99, or A/California/7/09 were [35S]-Met pulse-labeled and chased at 37°C before being detergent-lysed. HA species were precipitated with the StRAb 1F02 at 4°C and then analyzed by non-reducing SDS-PAGE and fluorography. (TIF) [file ppat.1004204.s002.tif]

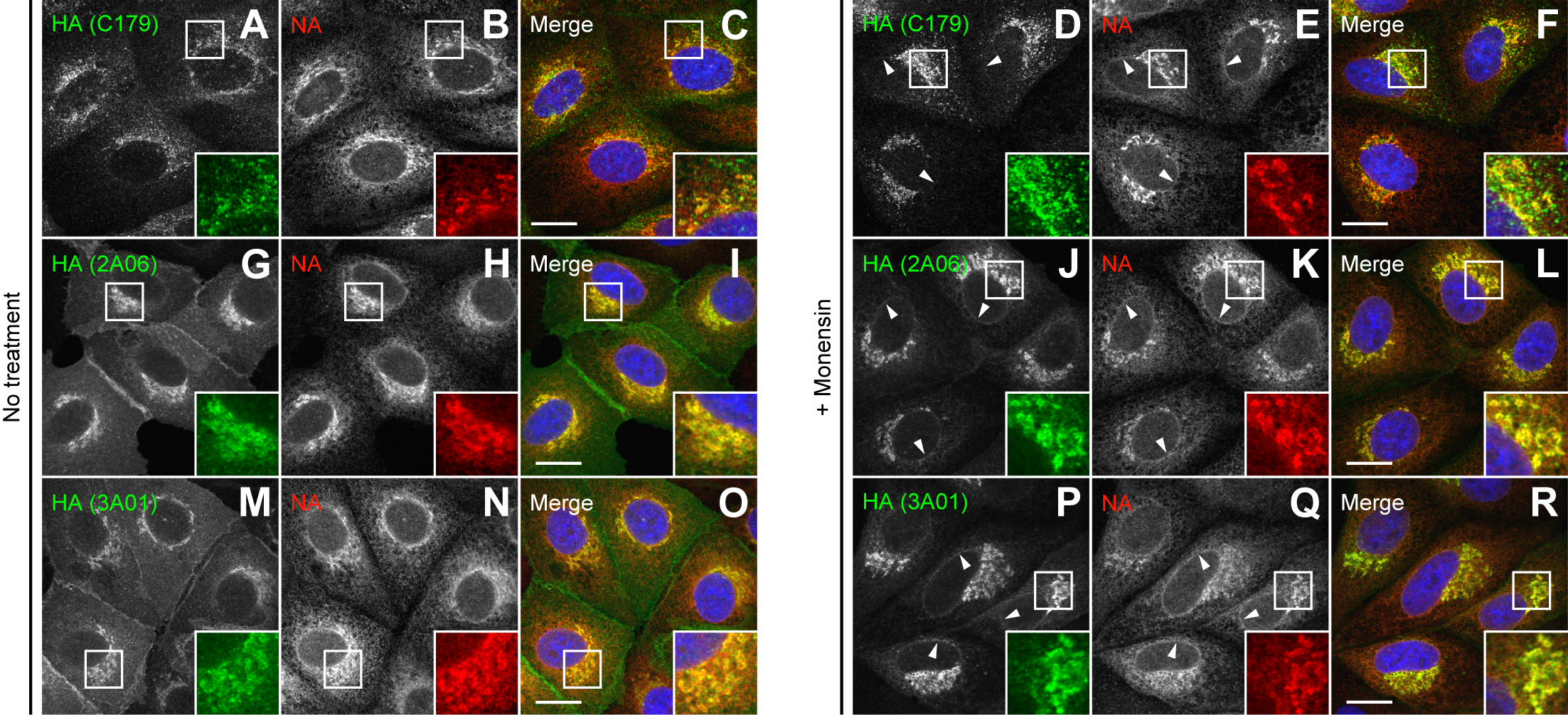

Supplement: Figure S3 — Reactivity of additional StRAbs to HA monomers and trimers assayed by immunofluorescence confocal microscopy. (A–R) MDCK cells were infected with IAV PR8 in the absence (no treatment) or presence of 10 µM monensin as described in Fig. 1, B–M. HA was labeled on fixed and permeabilized cells using the mouse StRAb C179 (A–F) or the human StRAbs 2A06 (G–L) and 3A01 (M–R) (green channel). NA was detected using rabbit pAbs (red channel). DNA was visualized using DAPI (blue channel). Stained cells were examined by fluorescence confocal microscopy. Bars: 10 µm. Arrowheads point NA co-localizing with HA monomers in the nuclear envelope (ER). (TIF) [file ppat.1004204.s003.tif]

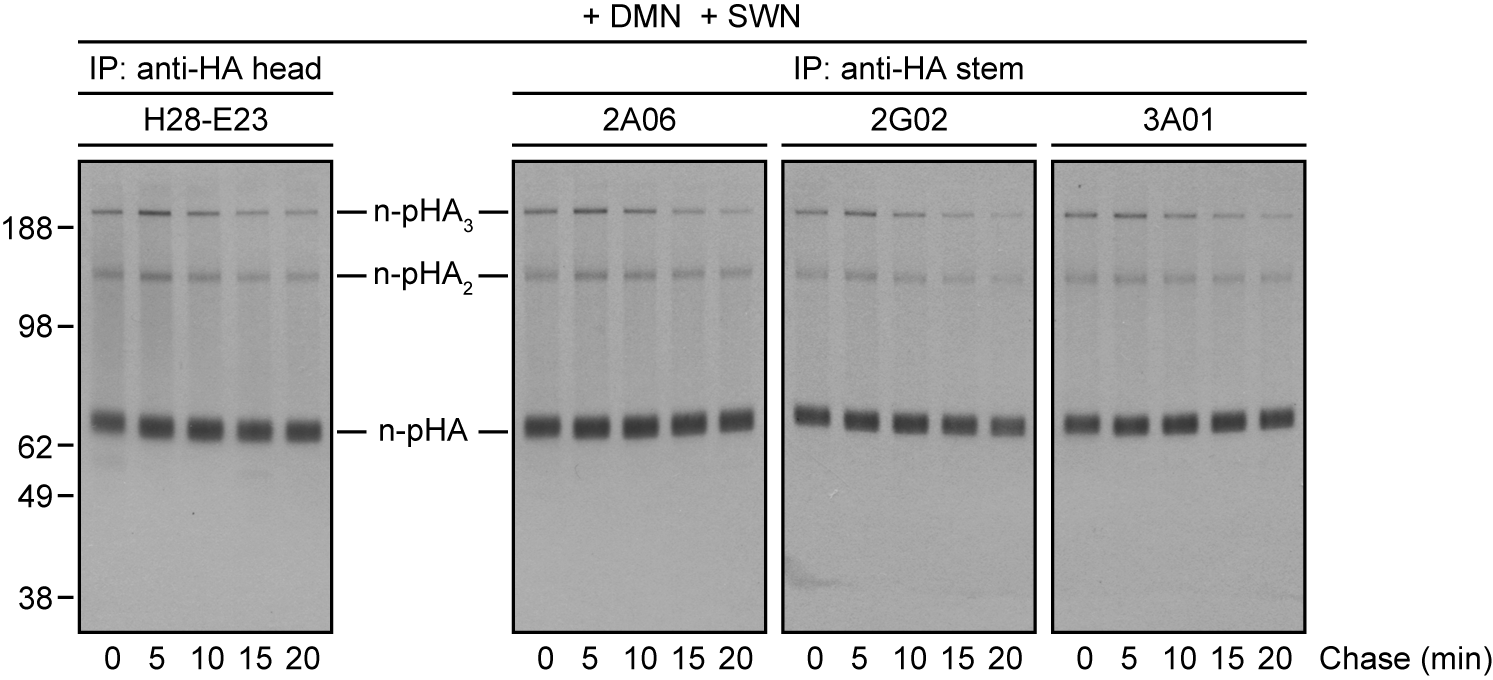

Supplement: Figure S4 — Inhibition of N -linked glycan processing allows proper StRAb binding to HA. IAV PR8-infected MDCK cells were treated with a mixture of DMN and SWN before being pulse-labeled with [35S]-Met and chased at 37°C in the continuous presence of the inhibitors as described in Fig. 3, B and C. Detergent cell extracts were incubated with the anti-HA head mAb H28-E23 (control) or the StRAbs 2A06, 2G02, and 3A01 at 4°C. Immunocollected proteins were resolved by SDS-PAGE under non-reducing conditions and visualized by fluorography. n-pHA: non-processed, glycosylated HA. (TIF) [file ppat.1004204.s004.tif]

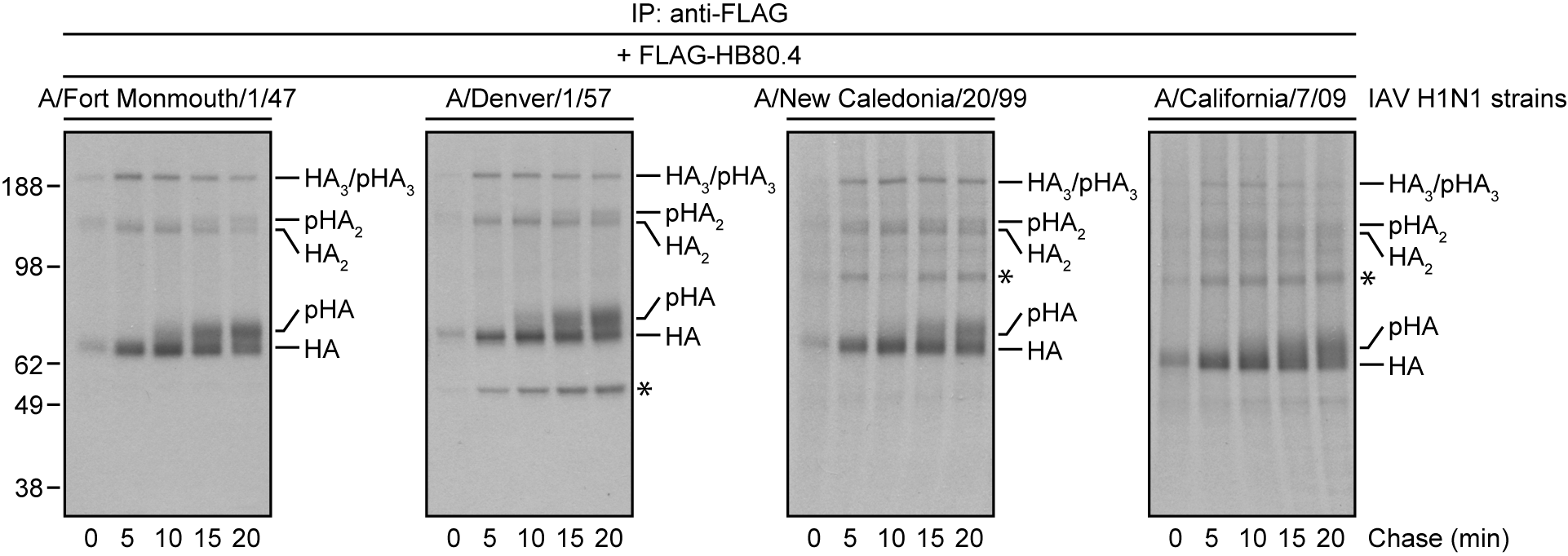

Supplement: Figure S5 — HB80.4 overcomes N -linked oligosaccharide shielding within the stem region of HAs from additional drifted human IAV H1N1 strains. MDCK cells infected with IAV A/Fort Monmouth/1/47, A/Denver/1/57, A/New Caledonia/20/99, or A/California/7/09 were labeled with [35S]-Met and chased at 37°C. Detergent cell lysates were incubated with FLAG-tagged HB80.4 at 4°C. HA species in complex with HB80.4 were precipitated with the anti-FLAG mAb M2 also at 4°C and then analyzed by non-reducing SDS-PAGE and fluorography. Asterisk: non-specific band; pHA: processed, glycosylated HA. (TIF) [file ppat.1004204.s005.tif]
